# New Data on Organization and Spatial Localization of B-Chromosomes in Cell Nuclei of the Yellow-Necked Mouse *Apodemus flavicollis*

**DOI:** 10.3390/cells10071819

**Published:** 2021-07-19

**Authors:** Tatyana Karamysheva, Svetlana Romanenko, Alexey Makunin, Marija Rajičić, Alexey Bogdanov, Vladimir Trifonov, Jelena Blagojević, Mladen Vujošević, Konstantin Orishchenko, Nikolay Rubtsov

**Affiliations:** 1 Institute of Cytology and Genetics, The Siberian Branch of the Russian Academy of Sciences, 630090 Novosibirsk, Russia; keor@bionet.nsc.ru (K.O.); rubt@bionet.nsc.ru (N.R.); 2 Institute of Molecular and Cellular Biology, The Siberian Branch of the Russian Academy of Sciences, 630090 Novosibirsk, Russia; rosa@mcb.nsc.ru (S.R.); vlad@mcb.nsc.ru (V.T.); 3 Wellcome Sanger Institute, Cambridge CB10 1SA, UK; alex@mcb.nsc.ru; 4 Institute for Biological Research “Siniša Stanković”, National Institute of Republic of Serbia, 11060 Belgrade, Serbia; marija.rajicic@ibiss.bg.ac.rs (M.R.); jelena.blagojevic@ibiss.bg.ac.rs (J.B.); mladenvu@ibiss.bg.ac.rs (M.V.); 5 Koltzov Institute of Developmental Biology, Russian Academy of Sciences, 119991 Moscow, Russia; a.s.bogdanov@idbras.ru; 6 Department of Genetic Technologies, Novosibirsk State University, 630090 Novosibirsk, Russia

**Keywords:** B-chromosomes (Bs), repetitive DNA, duplication clusters, speciation, 3D genome organization, confocal microscopy, chromosome territories (CT), microdissection

## Abstract

The gene composition, function and evolution of B-chromosomes (Bs) have been actively discussed in recent years. However, the additional genomic elements are still enigmatic. One of Bs mysteries is their spatial organization in the interphase nucleus. It is known that heterochromatic compartments are not randomly localized in a nucleus. The purpose of this work was to study the organization and three-dimensional spatial arrangement of Bs in the interphase nucleus. Using microdissection of Bs and autosome centromeric heterochromatic regions of the yellow-necked mouse (*Apodemus flavicollis*) we obtained DNA probes for further two-dimensional (2D)- and three-dimensional (3D)- fluorescence in situ hybridization (FISH) studies. Simultaneous *in situ* hybridization of obtained here B-specific DNA probes and autosomal C-positive pericentromeric region-specific probes further corroborated the previously stated hypothesis about the pseudoautosomal origin of the additional chromosomes of this species. Analysis of the spatial organization of the Bs demonstrated the peripheral location of B-specific chromatin within the interphase nucleus and feasible contact with the nuclear envelope (similarly to pericentromeric regions of autosomes and sex chromosomes). It is assumed that such interaction is essential for the regulation of nuclear architecture. It also points out that Bs may follow the same mechanism as sex chromosomes to avoid a meiotic checkpoint.

## 1. Introduction

In recent years, there has been a significant deepening and expansion of ideas about what constitutes the genome of a species. The application of whole genome sequencing has made a huge contribution, but it became evident that the same species genomes can vary on different levels, including the chromosomal one. Thus, a number of species have additional B chromosomes (Bs), in addition to the main chromosome set or A chromosomes (As). Bs can vary in number and size in some species. Another example of chromosome variability is the directed elimination of specific genomic regions in somatic cells: the phenomenon is known as “chromatin diminution” and was first described in the 1880s for *Ascaris megalocephala* [1,2]; later, several other species with chromatin diminution were described, including zebra finch, where large, mainly heterochromatic chromosomes that presented only in germ cells were found [3]. Recently, it was shown that such germline-restricted chromosomes (GRC) are probably presented in germ cells of all songbirds [4]. Sequencing the complete genomes of individuals with and without Bs [5,6], as well as microdissected Bs of a number of mammalian and insect species and the GRC of several bird species revealed the presence of additional sequences, which can be considered as copies of the “standard” genome genes [7,8,9,10,11], in some cases, together with adjacent areas [12,13]. It was also shown that at least some of the genes of the Bs and GRC are transcriptionally active [14,15]. The question of the location, timing and significance of the expression of these genes remains open.

Moreover, an integrated understanding of the genome structure should include not only a set of DNA sequences but also the dynamics of nucleotide and histone modifications in ontogenesis, the spatial arrangement of genomic elements and their dynamics through both organism development and the cell cycle. That is, the genome should be considered as a complex multicomponent highly organized structure, which is characterized by the variability of its individual parts, packaging of individual elements and their determined localization in the space of the interphase nucleus.

Bs, being a variable part of the genome, are of particular interest, since they sometimes demonstrate significant intraspecific variation in their number, size, and morphology and undoubtedly, they contribute to the architectonics of the interphase nucleus. Numerous studies have assessed the origin, gene composition, structural organization, and evolution of Bs, as well as their transcriptional activity and influence on the adaptive potential of their carriers. Recently, the origin, evolution, behavior in mitosis and meiosis, and DNA composition of the Bs in mammals was discussed [16,17,18]. In brief, it was concluded that: (i) most of studied Bs of mammals were derived from pericentromeric A chromosome regions followed by DNA transposition and amplification, but another mechanism might have included amplification of repeats leading to formation of regions enriched for repetitive DNA and loss of euchromatic regions located between them [9,19,20]; (ii) Bs consist mainly of repetitive sequences homologous to repeats located in the As, but extended regions carrying some genes were also revealed in some Bs, furthermore, the cases of its transcription were described; (iii) in mitosis Bs are similar to the As, however, intraindividual diversity of the B chromosome number in some analyzed samples allows us to suggest an increased instability of Bs in cell division [21,22]; (iv) behavior of the Bs in meiosis is even more mysterious, as they demonstrate different patterns of replication, conjugation, and segregation [18,23].

Still, Bs remain mysterious elements of the karyotype, leaving a lot of questions open [8,16]. Studying the influence of Bs on the three-dimensional organization of the nucleus and the process of meiosis can make a significant contribution to our understanding of localization of genome elements in various compartments of the nucleus, the peculiarities of chromosome conjugation and their recombination in meiosis.

Mammalian chromosomes are characterized by the clear structural organization: pronounced G-, C-, and R-bands differ in the composition of their DNA, the types and density of genes, the replication timing, and transcriptional activity. This structure made it possible to elevate problems of 3D-organization of the interphase nucleus in mammalian cells to a fundamentally new level. Numerous studies have shown nonrandom localization of C-, G-, and R-segments, telomeric and centromeric regions of chromosomes in the interphase nucleus [24,25,26,27]. These studies extensively used three-dimensional fluorescent in situ hybridization (3D-FISH) followed by 3D microscopy. A significant contribution to modern ideas on the spatial organization of the nucleus was made by experiments using chromosome conformation capture (from 3C to 5C) technologies, which allowed to identify topologically associated domains [28,29] and to demonstrate their evolutionary conservation [30].

However, not all methods that have shown their high efficiency in studies of localization of individual chromosomes and their regions in the interphase nucleus turned out to be as effective when applied to Bs. Enrichment of Bs with repetitive DNA sequences [8], the laboriousness of sequencing them [8,31], and insufficient knowledge about the genomes of most species with Bs either significantly complicate or make it almost impossible to apply most of these methods. Using DNA probes specific to different regions of Bs, which allowed to get clear fluorescent in situ hybridization (FISH) signals, are more of a lucky exception than the rule [12,32]. An additional problem that complicates studying Bs is their intraspecific diversity, both in size and DNA composition [33]. A detailed and versatile description of individual Bs would be correct if it concerned not only their morphology but also the DNA composition of their individual regions, including fragments, homologous to the unique sequences of the main genome. Unfortunately, in most studies, characterization of Bs is limited to describing the distribution of a small set of repeated sequences, which are homologous to rDNA, telomeric DNA, DNA of C-positive regions of A chromosomes [8,9,34,35].

The Bs of mice of the genus *Apodemus* (the yellow-necked mouse *Apodemus flavicollis* Pallas, 1811 and the East-Asian (Korean) mouse *A. peninsulae* Thomas, 1907) are an example of the most thoroughly studied mammalian additional chromosomes. A large number of animals from different geographic regions were studied using both routine and molecular cytogenetic methods of chromosome analysis [19,36,37]. Moreover, microdissected DNA libraries of several Bs of *A. flavicollis* and *A. peninsulae* were sequenced that revealed regions, homologous to those in the house mouse genome [11]. Localization of Bs in the nuclei of fibroblasts and spermatocytes of *A. peninsulae* was assessed using 3D-FISH and confocal microscopy, and the influence of Bs on the structural and functional organization of the nucleus was discussed [38]. The analysis of the localization of *A. flavicollis* Bs is of particular interest, since, unlike *A. peninsulae* chromosomes, they contain rather large C-negative regions [39,40]. Here, we aimed to clarify the organization of Bs in *A. flavicollis* and analyze their localization in the interphase nucleus. We applied 2D and 3D-FISH with DNA probes obtained from microdissected Bs and the pericentromeric regions of *A. flavicollis* autosomes [39,41]. The results of the analysis were used to clarify the Bs origin, evolution, and possible influence on the standard genome of *Apodemus* mice.

## 2. Materials and Methods

### 2.1. Specimens

Fifteen specimens of *A. flavicollis* were trapped in natural populations of the Russian Federation, the Republic of Belarus, and Republic of Serbia. Furthermore, to compare the spatial organization of interphase nuclei in different mouse species, which have Bs in their karyotypes, we included in the analysis two specimens of the East-Asian mouse *A. peninsulae* (Table 1). These species were described earlier in the study devoted to spatial organization of interphase nuclei of *A. peninsulae* [38]. All animals were captured with Longworth traps. The animals captured in Serbia were treated according to standard procedures (Directive 2010/63/EU of the European Parliament and the Council of 22 September 2010 on the protection of animals used for scientific purposes). The mice captured in Russia and Belarus were treated according to protocols approved by the Animal Care and Use Committee of the Institute of Cytology and Genetics SB RAS (Novosibirsk) (protocol #45/2 from 10.01.2019). All experiments were performed according to the Declaration of Helsinki. No additional permits are required for research on non-listed species in Russia, Belarus, and Serbia. Metaphase chromosomes of thirteen specimens were analyzed earlier in the study devoted to clarification of the origin of the Bs in *A. flavicollis* [41].

### 2.2. Cell Cultures

We used primary fibroblast cultures established from four animals captured in natural populations of Central Serbia [41]. Cells were cultured according to standard protocol [40,42,43]. Three passages were performed before the preparation of metaphase chromosomes and cell nuclei for the 3D-FISH study.

### 2.3. Bone Marrow Suspension Preparations

Cell suspensions were prepared from bone marrow using methods similar to the ones described earlier ones [44]. Each mouse was given an intraperitoneal injection of colchicine (Sigma-Aldrich, Saint Louis, MO, USA) in physiological saline at the dose of 2 µg/g body weight. The mice were euthanized in one hour after this treatment; femurs were dissected and the bone marrow cells flushed out with the sterile phosphate-buffered saline, pH 7.4. The cells were gently pelleted and subsequently swollen in the hypotonic solution (75 mM KCl). They were fixed in methanol-acetic acid (3:1).

### 2.4. Metaphase Chromosome Preparation and Karyotyping of Animals

Metaphase chromosome preparation from cultured fibroblasts or bone marrow cells was carried out as described earlier [41]. Chromosomes were stained with DAPI. The number of Bs in specimens was calculated and described in at least twenty metaphase spreads per individual.

### 2.5. DNA-Probe Generation from Pericentromeric Region of the Large Autosome of A. flavicollis Karyotype

DNA-probe from the pericentromeric region (PCPAflaCEN) of the largest autosome of *A. flavicollis* specimen #1851 was generated by metaphase chromosome microdissection followed by a degenerate oligonucleotide-primed polymerase chain reaction (DOP-PCR) with Sequenase 2.0 (ThermoFisher Scientific, Waltham, MA, USA) according to standard protocol [39,45,46]. Briefly, the chromosome stained with Giemsa (Sigma-Aldrich, Saint Louis, MO, USA) was dissected with an extended glass needle controlled with micromanipulator MR (ZEISS, Jena, Germany) ) on an inverted microscope AXIOWERT10; one copy of the pericentromeric region of the largest autosome was transferred into the extended and siliconized tip of Pasteur pipet with 40 nL collection solution, containing 30% glycerol, 10 mM Tris-HCl (pH 7.5), 10 mM NaCl, 0.1% SDS, 0.1% Triton X-100, 500 μg/mL proteinase K (Boehringer Mannheim, Indianapolis, IN, USA). Dissected material was treated for two hours in a moist chamber at 60 °C and then transferred into 5 µL of PCR mixture for DOP-PCR, which was performed as described [46,47]. DNA labeling was carried out with 20 additional cycles of PCR performed with an additional fluorescent labeled nucleotide Alexa Fluor 488-5-dUTP (Invitrogen, Waltham, MA, USA) [46,48].

### 2.6. Whole Chromosome Paints derived from the B-Chromosomes of A. flavicollis

Whole Chromosome Paints (WCPBs) were derived from two Bs of the *A. flavicollis* female #24985 (WCP24985aB and WCP24985bB), one of Bs of the male #3727 (WCP3727B), one of Bs of females #3980 (WCP3980B), #3977 (WCP3977B) and #26368 (WCP26368B). Probes were generated earlier by metaphase chromosome microdissection and DNA amplification using a GenomePlex Single Cell Whole Genome Amplification Kit (WGA4) (Sigma-Aldrich, Saint Louis, MO, USA) according to the manufacturer’s protocol [41], labeled with TAMRA-5-dUTP (Roche) and used for 2D- and 3D-FISH.

### 2.7. Preparation of 3D-Preserved Interphase Nuclei for FISH Analysis

#### 2.7.1. Suspension FISH (S-FISH)

Partial chromosome painting with the use of the probe PCPAflaCEN and whole Chromosomes Paints were applied together in suspension-FISH (S-FISH) as previously reported [49,50,51,52] with some modifications. In short, the entire FISH procedure was performed on cell suspension, and the interphase nuclei were placed on a polished concave slide at the final step of the procedure, just before the evaluation. It was shown before that it is possible to do 3D analyses on totally spherical interphase nuclei using S-FISH [52]. The main steps of the S-FISH technique included: pepsin treatment (i.e., 475 μL H_2_O, 25 μL 0.2 N HCl, 0.005% pepsin), simultaneous denaturation of DNA in interphase cells and DNA-probe at 95 °C without suppression of repetitive sequences, hybridization overnight at 37 °C, washing in 0.4 SSC and 4 × SSC/0,2% Tween, detection and counterstaining in 0.5% DAPI-Vectashield (Vectashield; Vector, Burlingame, CA, USA) [51].

#### 2.7.2. 3D-FISH of Fibroblasts

Fibroblasts were seeded onto 60 × 24 mm coverslips in quadriperm plates in DMEM, which was supplemented with 10% fetal calf serum (FCS) and cultivated in the CO_2_ incubator (5% CO_2_ at 37 °C) until a subconfluent monolayer was formed. Then coverslips were briefly rinsed in PBS and fixed at room temperature in freshly prepared 4% paraformaldehyde for 10 min. Permeabilization of nuclei was performed with 0.5% Triton X-100 in PBS for 15 min, and then in PBS with 20% glycerol for 30 min, repeated freezing/thawing in liquid nitrogen four times, and incubation in 0.1 mol/L HCl (10 min) as it was described [53]. A minor modification to the protocol was including RNAase treatment (100 μg/mL in 2 × SSC) at 37 °C for 60 min, was included in the treatment. Procedures were carried out without drying of the cells. Slides were kept at 4 °C in 50% formamide/2 × SSC until hybridization. The labeled probes were dissolved in hybridization mixture (50% formamide, 10% dextran sulfate, 2 × SSC, 0.01% NP-40), loaded on the slide with pretreated cells, covered with a coverslip (24 × 24 mm), and sealed with rubber cement. Then DNA of nuclei and probes was denatured on a hot-block of Thermomixer comfort, Eppendorf (Hamburg, Germany) at 73 °C for 5 min. Hybridization was performed for two days in humid boxes at 37 °C. Post-hybridization washing was performed in 0.1 × SSC at 60 °C and 4 SSC/0.1% NP-40 solutions at 45 °C for 5 min. Then nuclear DNA was counterstained with DAPI and mounted in Invitrogen Prolong Gold Antifade, Invitrogen (Waltham, MA, USA).

#### 2.7.3. 2D- and 3D-Microscopy

Two-dimensional (2D)-microscopy was carried out with an Axioskop 2 Plus microscope (Zeiss, Jena, Germany) equipped with a CCD camera and filter sets (ZEISS #49, #10, #20). ISIS5 image-processing package of MetaSystems GmbH (METASystems GmbH, Altlußheim, Germany) was used to capture and process microscopic images. Confocal microscopy was performed using LSM510META confocal system (ZEISS) with a Diode 405 nm, Argon 458, 477, 488, 514 nm, Helium/Neon 543 nm, and 633 nm laser lines; filters BP 445-450, BP 420-480 IR, BP 445-450, BP 505-570, and LP 650 based on AxioObserver Z.1 microscope (Zeiss, Jena, Germany, ×63 oil immersion objective with a numerical aperture of 1.4) were applied. The thickness of the optical slice was 0.6 µm. The images were analyzed using LSM Image Browser (Zeiss, Jena, Germany) and ZEN 2009 software (Zeiss, Jena, Germany). Microscopy was performed at the Inter-institutional Shared Center for Microscopic Analysis of Biological Objects (The Institute of Cytology and Genetics, SB RAS, Novosibirsk, Russia).

### 2.8. Evaluation

Images of 3D-preserved interphase nuclei were captured using a Zeiss Axioskop 2 Plus microscope and analyzed by the Zen-2012 (Zeiss, Jena, Germany) software. At least 20 interphase nuclei were acquired and processed per specimen. Interphase nuclei were stained with DAPI to define the nuclear boundaries [54]. For the 3D-evaluation, the position of homologous chromosomes and distance between them were determined. In the analysis, internal and external compartments (i.e., the center and the periphery) were determined in interphase nuclei with spherical shapes. Thus, analyzed chromosomes could be allocated to either in the center or on the periphery of a nucleus. Similar to results described in the previous publication [50], the relative positions of the studied chromosomes to each other were recorded in the center, on the periphery or both. A chromosomes located on the borderline between the compartments were classified as central or peripheral in relation to where the majority of the chromosome body was located. Statistical analysis was performed using Mann–Whitney U test. Statistical significance was defined as *p* < 0.05.

The nuclei of in vitro cultured fibroblasts have a pronounced feature of their morphology: the length of the nucleus is significantly greater than its width, while the height is low. Usually, chromosomes contact with nuclear boundaries both in the lower and the upper sides [55,56]. In this case, it makes no sense to define the inner and outer compartments of a nucleus when analyzing the localization of chromosomal territories. However, considering the position of chromosomes relative to the nuclear envelope, we distinguished two types of its localization: (1) in the lateral, upper and lower contact with the nuclear envelope; (2) only in the upper and lower contact with shell of the nucleus. In the first case, the area of contact with the nuclear envelope can be 1.5-times larger than in the second case.

## 3. Results

### 3.1. DNA Homology in Pericentromeric Regions of A- and B-Chromosomes in A. flavicollis

#### FISH of B-specific DNA Probes with *A. flavicollis* Chromosomes

Previously, we characterized the details of repetitive DNA distribution using high-throughput sequencing [20] and FISH [41]. Using the probes, obtained earlier by microdissection of Bs, autosomes, and sex chromosomes from karyotypes of *A. flavicollis* individuals [39,41,57,58], we continued the analysis of FISH patterns, which were produced by in situ hybridization of the probes with metaphase chromosomes of yellow-necked mice from Serbia, Belarus, and Russia, including specimens from several additional localities (mainly, from the North-Eastern part of the species range) (Table 1).

**Figure 1:**
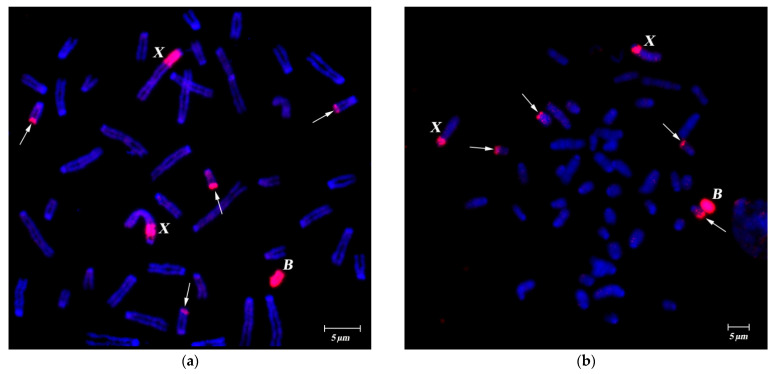
FISH of B-specific Whole Chromosome Paint (WCPB) (red signal) onto metaphases of females with one B chromosome from different populations of *A. flavicollis*. (**a**) WCP3977B probe (from a specimen from Petnica, Serbia) hybridized onto metaphase plate from fibroblast cell culture of female #3980 (Orašac, Serbia); (**b**) WCP3980B probe (from a specimen from Orašac, Serbia) onto a metaphase plate from bone marrow cells of female #26368 (Ulyanovsk region, Russia). Bs and X chromosomes are marked by corresponding letters; arrows indicate heterochromatic blocks on four small autosomes. DAPI total chromosome staining is blue. Scale bar 5 µm.

**Figure 2:**
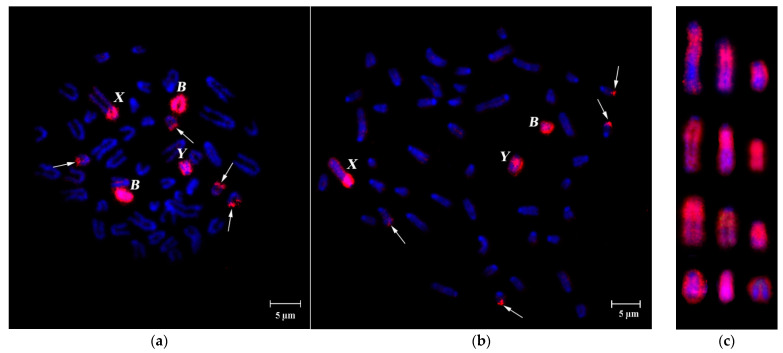
FISH of B-specific Whole Chromosome Paint (WCP3980B) (from specimen from Orašac, Serbia, red signal) onto metaphases with Bs from bone marrow cells of A. flavicollis males. (**a**) Metaphase plate of specimen #3687 (2B) (Milošev Do, Serbia); (**b**) metaphase plate of specimen #24944 (B) (Minsk region, Republic of Belarus); (**c**) a gallery of Y chromosomes with FISH signals from different metaphase spreads of four individuals. Every row in (**c**) represents a single specimen. Bs, X and Y-chromosomes are marked by corresponding letters; arrows indicate heterochromatic blocks on four small autosomes. DAPI total chromosome staining is blue. Scale bar 5 µm.

**Figure 3:**
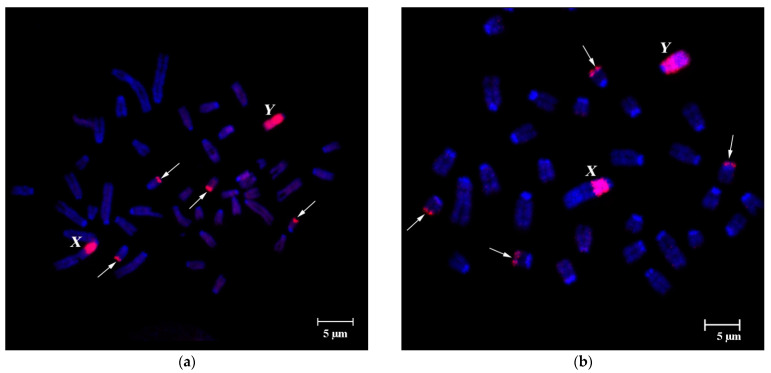
FISH of B-specific Whole Chromosome Paint (WCPB) (red signal) onto metaphases of males without Bs from fibroblast cell culture of *A. flavicollis* individuals. (**a**) WCP3977B probe (from a specimen from Petnica, Serbia) hybridized onto metaphase plate of specimen #3979 (Orašac, Serbia); (**b**) WCP3980B probe (from a specimen from Orašac, Serbia) hybridized onto metaphase plate of specimen #3978 (Orašac, Serbia). Bs, X and Y-chromosomes are marked by corresponding letters; arrows indicate heterochromatic blocks on four small autosomes. DAPI total chromosome staining is blue. Scale bar 5 µm.

FISH of specific B-chromosome probes (WCP24985B, WCP26368B, WCP3980B, WCP3977B and WCP3727B) showed similar affinity to Bs, the pericentromeric region of sex chromosomes and the subtelomeric region of four small autosomes, despite geographically distant sample origins both in latitudinal and longitudinal directions (Figure 1a,b, Figure 2a–c and Figure 3a,b). The strong signal from hybridization of these probes with different male metaphases confirmed high homology of all tested WCPBs to the pericentromeric region of sex chromosomes and lower homology with the rest of the Y chromosome (Figure 2a–c). However, we observed here that FISH-signals on the Y chromosome significantly differ in their intensity between individuals (Figure 2 and Figure 3a–c).

Hybridization of B-specific probes to preparations of male metaphases without Bs, also confirmed the similarity of Bs to the pericentromeric region of sex chromosomes and the difference of FISH-signal intensity between individuals (Figure 3).

### 3.2. FISH of DNA Probe Generated from the Pericentromeric Region of a Large Autosome of A. flavicollis

**Figure 4:**
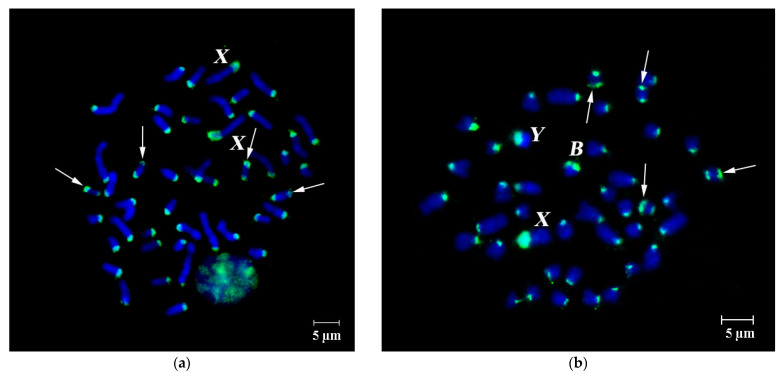
FISH of microdissected DNA probe (PCPAflaCEN, green) generated from the pericentromeric region of a large autosome on metaphase chromosomes of: (**a**) the same *A. flavicollis* specimen, from which the probe was derived (no Bs, female #1851 from Saint-Petersburg region, Russia); (**b**) another *A. flavicollis* specimen (with one B, male #24448 from Novgorod region, Russia). Arrows indicate the signal from PCPAflaCEN probe in telomeric regions of two pairs of small autosomes; B chromosome and sex chromosomes are marked by corresponding letters. DAPI chromosome staining in blue. Scale bar 5 µm.

FISH of microdissected DNA probe generated from the pericentromeric region of a large autosome of *A. flavicollis* female #1851 without Bs (PCPAflaCEN) labeled pericentromeric C-positive regions of varying size on autosomes of *A. flavicollis* (Figure 4).

Detection of homologous DNA in these chromosomal regions entirely agrees with earlier studies [39]. Previously, considerable size variation was shown for C-positive regions of X chromosomes, containing DNA, which is homologous to DNA of Bs [8,9,33,39]. Here, we also detect considerable size variation in the Y chromosome region, containing repeats, homologous to either DNA of Bs (Figure 2 and Figure 3) or DNA of pericentromeric regions of autosomes (Figure 4a,b). Fluorescent signal from PCPAflaCEN DNA probe was localized at the subtelomeric regions of B chromosomes but not at the centromeric regions of Bs (Figure 4b).

### 3.3. Dual-Color FISH of WCPBs and PCPAflaCEN Probes on Metaphase Plates of A. flavicollis

**Figure 5:**
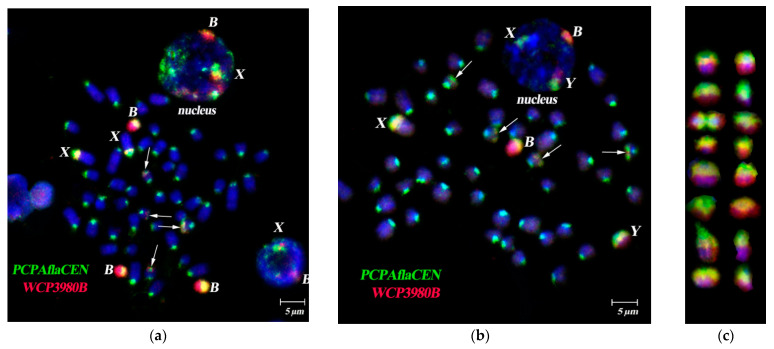
Simultaneous FISH of the B-specific Whole Chromosome Paint (WCP3980B, red signal) and PCPAflaCEN probe, obtained from the pericentromeric region of a large autosome (green signal) on metaphase chromosomes of: (**a**) a female with three Bs (#3854, Misača, Serbia); (**b**) male with one B (#24944, Minsk region, Republic of Belarus); (**c**) a gallery of Y chromosomes with FISH signals from different metaphase spreads of four specimens. Every two rows in (**c**) represent a single specimen. Arrows indicate FISH-signals from both probes in subtelomeric regions of two pairs of small chromosomes. Bs, X and Y chromosomes are marked by letters B, X and Y, respectively, on metaphase plates and nuclei. DAPI staining is blue. Scale bar 5 µm.

Simultaneous use of two probe types (i.e., any B-specific probe and the PCPAflaCEN probe) generated partly overlapping signals in dual-color FISH on Bs and sex chromosomes (Figure 5). FISH of the PCPAflaCEN probe with metaphase plates labeled pericentromeric C-positive regions of varying size on autosomes and sex chromosomes, in accordance with earlier published data [39]. The region immediately adjacent to the centromere of the X chromosome, was mostly comprised of repeats homologous to the pericentromeric regions of the autosomes. The rest of the C-positive pericentromeric block also contained typical for Bs repeats (Figure 1 and Figure 5a–c).

On the Y chromosome, signal patterns of DNA probes obtained from the B chromosome (WCP3977B, WCP3980B) and autosomal pericentromeric regions also significantly differed between individuals (Figure 2, Figure 3, Figure 4 and Figure 5). FISH signals from the PCPAflaCEN probe were revealed in distal regions of Bs (Figure 4b and Figure 5). Previously, only relatively small signals of low intensity were detected on the Y chromosomes of the studied animals after FISH with PCPAflaCEN. However, in some animals involved in this study, the Y chromosomes consisted of regions enriched with repeats of autosomal pericentromeric heterochromatin (in the proximal part of the Y chromosome) (Figure 4b) or repetitive DNA of Bs (in the distal part) (Figure 2, Figure 3 and Figure 5b). At the boundary of these regions, the signals of both DNA probes were combined (Figure 4b). Unfortunately, it is not possible to determine whether it is due to the presence of regions that include both types of repeats, or it results from DNA spreading during the preparation of metaphase chromosomes. The phenomenon of detecting regions with combined signals became well known as a result of the widespread use of 24-color FISH for detecting chromosome translocations in human chromosomal pathologies [59,60,61]. Variations in size and DNA composition of the C-positive X chromosomal region and Y chromosome indicate the ongoing processes of DNA amplification, characteristic for Bs, and the reorganization of these regions.

Taking into account the discrepancy in patterns of FISH-signals, generated with the use of B-specific probes and PCPAflaCEN probe, as well as a quite considerable size of dissected chromosome regions, we can propose that the partial signal overlap from two probes in two-color FISH is due to the existence of both different types of DNA repeats in Bs and pericentromeric heterochromatin of sex chromosomes. Therefore, Bs are heterogeneous in the content of DNA repeats. However, finding only trace signals from B-specific DNA probes in the pericentromeric regions of autosomes indicates that the number of copies, homologous to DNA repeats of pericentromeric autosome heterochromatin, seems to be very small in Bs. Consequently, the minor signals from B-specific probes were masked by much more multiple repeats, originated from C-positive autosome region.

Thus, using both probes demonstrates the most similarity in composition of DNA repeats in Bs and pericentromeric heterochromatic regions of sex chromosomes.

### 3.4. FISH Signal Distribution of WCPBs and PCPAflaCEN Probes in Interphase Nuclei of A. flavicollis

**Figure 6:**
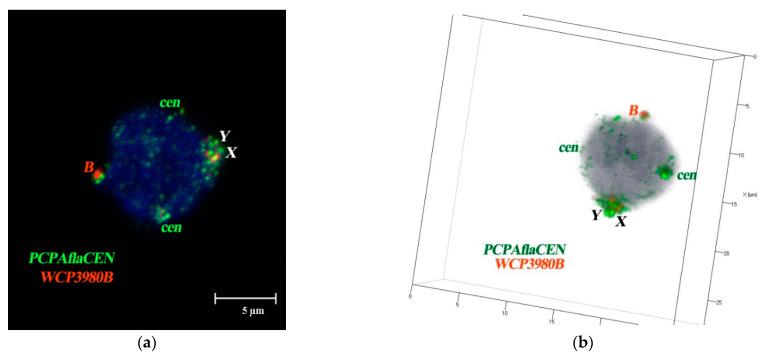
S-FISH of B-specific Whole Chromosome Paint (WCP3980B, red signal) and PCPAflaCEN probe (green signal) with the interphase nuclei of bone marrow cells of *A. flavicollis* male #24448 (1B, Novgorod region, Russia). (**a**) Maximum intensity projection; (**b**) three-dimensional reconstruction. Bs, X and Y chromosomes are marked by corresponding letters, “*cen”* indicates autosomal C-positive regions; DAPI staining is blue; magnification is 63×. Conditions of scanning are the following: laser lines 405, 488; filters BP 420-480 IR and BP 505-570. Thickness of optical slice is 0.6 µm.

As in the case of two-color FISH, applied to metaphase chromosomes, the territories of Bs in the interphase nuclei were characterized by an intense WCP signal from the B-specific WCP3980B probe and also a weaker signal from PCPAflaCEN probe in the distal regions of Bs. FISH signals in the euchromatic regions of A chromosomes did not exceed the background level. The pericentromeric C-positive autosomal regions were characterized by the FISH signals from the PCPAflaCEN probe only. The FISH signal from the B-specific DNA probe on A-chromosomes almost did not differ from the background (Figure 5 and Figure 6).

The territory of the Y chromosome in the interphase nuclei consisted of two separate regions, which were detected by FISH with either the PCPAflaCEN probe (proximal region) or the WCP3980B probe (distal region). At the boundary of these areas, there was a zone characterized by the presence of signals from both DNA probes. The territory of the C-positive X chromosome region also differed from the territories of Bs: its main part was stained with both the PCPAflaCEN probe and B-specific probe, while a small proximal region was stained with the PCPAflaCEN probe only. Such features of 3D-FISH of C-positive regions of autosomes, X, Y chromosomes and Bs made it possible to carry out their reliable identification in the interphase nuclei (Figure 5b and Figure 6).

### 3.5. Spatial Organization of Interphase Nuclei in Bone Marrow Cells of A. flavicollis

In an interphase nucleus, 3D-FISH did not reveal large-scale associations between C-positive regions of A chromosomes. Larger heterochromatin regions of sex chromosomes were usually not in association with each other. Their association with the pericentromeric regions of the autosomes could remain unrevealed since the C-positive regions of the sex chromosomes contain DNA repeats identical to the pericentromeric regions of the autosomes, as it was aforementioned.

**Figure 7:**
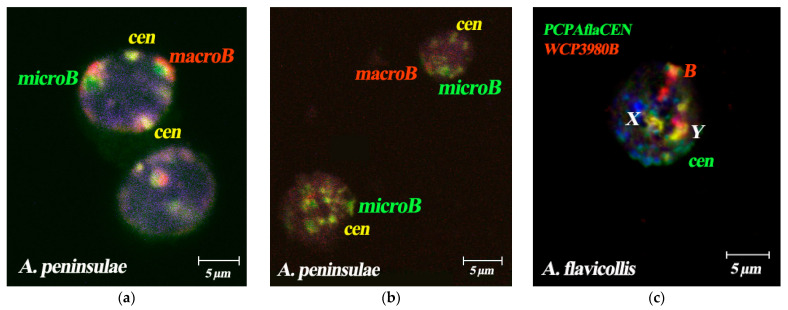
Maximum intensity projection of laser scanning microscopy through *Apodemus peninsulae* (**a**,**b**) and *A. flavicollis* (**c**) interphase nucleus with the Bs and centromere FISH signals. (**a**,**b**) Section showing association of B arm specific repeats type 1 (“*macro*” B, middle size, red), B arm specific repeats type 2 (“*micro B*”, small size, green) and pericentromeric regions (“*cen*”, yellow) in the interphase nuclei of spermatocytes of *A. peninsulae* specimen # 5 (male with 6B, Novosibirsk region, Russia) and *A. peninsulae* specimen # 9 (male with micro-6B, Krasnoyarsk district, Russia); (**c**) S-FISH of WCP3980B probe (red signal) and PCPAflaCEN probe (green signal) with the interphase nuclei of bone marrow cells of *A. flavicollis* male #24448 (1B, Novgorod region, Russia). Bs, X and Y chromosomes are marked by corresponding letters. Nuclei were stained with DAPI (blue).

Bs were mainly located at the periphery of the nucleus, without an association with A-chromosomes. Three-dimensional analysis of spherical nuclei showed that territories of Bs were intensely stained with the B-specific WCP3980B probe and in all cases were adjacent to the nuclear membrane; the pattern was analogous in *A. flavicollis* (Figure 6a,b) and *A. peninsulae* (Figure 7a–c). The main difference in the localization of the B chromosomes of the two compered species was that the Bs of *A. flavicollis* were located separately from the sex chromosomes (Figure 7c), while the B chromosomes of *A. peninsulae* showed a tendency to form a common compartment with a sex bivalent (Figure 7a,b). Similar results for different species were published earlier [38,54].

### 3.6. Spatial Organization of Fibroblast Interphase Nuclei with Bs

For all studied chromosomes, we registered both the lateral contacts with the nuclear membrane and the absence of such contacts. The proportion of contacts with the lateral nuclear membrane was 21.05% for Bs and 12.16% for X chromosomes. For all chromosomes and chromosomal regions involved in the study, their localization both with and without contacts with the lateral membrane was observed. However, all Bs and the C-positive regions of the X chromosomes were preferentially localized in the upper and lower contacts with the nuclear membrane, but without the lateral contacts (Figure 7a–c). In this study, we did not identify associations between pericentromeric regions of autosomes, Bs, and the C-positive regions of the X chromosomes. Problems with identification of pericentromeric regions of autosomes that could form an association with one of the sex chromosomes or the B chromosome do not allow us to conclude that such associations are absent.

**Figure 8:**
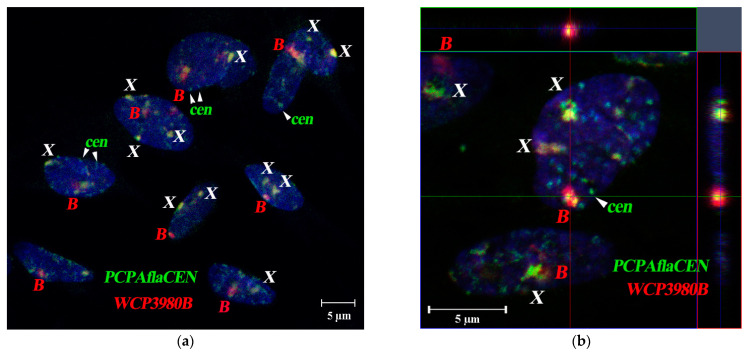
The 3D-FISH of the B-specific probe (WCP3980B, red signal) and the probe to pericentromeric heterochromatin of A-chromosomes (PCPAflaCEN, green signal) in the interphase nuclei of fibroblast from females with one B #3980 (Orašac, Serbia). (**a**) Group of fibroblast nuclei; (**b**) orthogonal projection, demonstrating the location of the Bs (red) and pericentromeric heterochromatin regions of As (green) in the interphase nuclei of fibroblasts of *A. flavicollis*. The blue, green and red lines indicate the location of axial, frontal, and sagittal optical sections, respectively. The axial optical section is in the center (xz), the frontal one is on top (xy), the sagittal one is on the right (yz). Co-localization of FISH-signals from B-specific probe and the PCPAflaCEN probe on X chromosomes (yellow). Bs and X chromosomes are marked by corresponding letters; cen—point to autosomal C-positive regions; DAPI staining is blue. Scale bar 5 µm.

**Figure 9:**
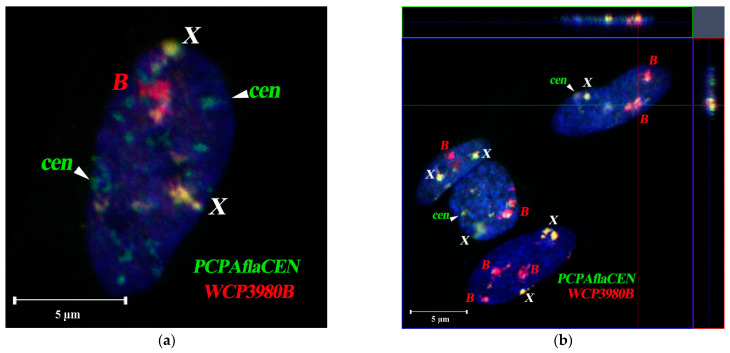
The 3D-FISH of the B-specific probe (WCP3980B, red signal) and the probe to pericentromeric heterochromatin of A-chromosomes (PCPAflaCEN, green signal) in the interphase nuclei of fibroblasts from female #3854 (Misača, Serbia) with three Bs. (**a**) Group of fibroblast nuclei; (**b**) orthogonal projection, demonstrating the location of the Bs (red) and pericentromeric heterochromatin regions of As (green). The blue, green and red lines indicate the location of axial, frontal, and sagittal optical sections, respectively. The axial optical section is in the center (xz), the frontal one is on top (xy), the sagittal one is on the right (yz). Co-localization of FISH-signals from B-specific probe and the PCPAflaCEN probe on X chromosomes is revealed as yellow signal. Bs and X chromosomes are marked by corresponding letters; cen—point to autosomal C-positive regions; DAPI staining is blue. Scale bar 5 µm.

We compared the chromosomal territories of Bs and sex chromosomes using confocal microscopy of 79 interphase fibroblast nuclei of *A. flavicollis* female #3980 with one B-chromosome (Orašac, Serbia) (Table 1, Figure 8a,b). For each chromosomal territory, we examined the association between B and X chromosomes, as well as the localization of both X chromosomes. The association of the C-positive region of the X chromosome and the B chromosome was observed in 13.8% of cases, while interaction between the two X chromosomes was detected only in 5% of cases. In nuclei of *A. flavicollis* female #3854 (Misača, Serbia), containing three Bs (Table 1, Figure 9), the proportion of associations of two or three Bs was 17.8%, the same association frequency (17.8%) was observed in the case of a close arrangement of B and X chromosomes. The proportion of associations of both X chromosome homologs increased to 9.5% in the specimen (Figure 9a,b).

Regardless of the degree of associations, both Bs and C-positive regions of sex chromosomes are localized at the periphery of the nuclei.

## 4. Discussion

### 4.1. The Origin of Bs in A. flavicollis

The use of probes which were derived by microdissection of whole Bs and pericentromeric heterochromatin region of the long autosome from karyotypes of yellow-necked mice allowed us to identify Bs as well as the sex chromosomes by the FISH approach. The Bs and the sex chromosomes demonstrated the distinctive signals, especially in the two-color FISH. As the general pattern of signals was constantly reproduced in specimens from very distant localities, it seems to be strongly specific for the whole species. Therefore, Bs may have a common origin and evolution in yellow-necked mice. They are mostly comprised of repetitive DNA, homologous to heterochromatin regions of sex chromosomes. This finding is in good agreement with the assumption that the Bs of *A. flavicollis* might originate from one of the sex chromosomes [41]. To establish themselves, proto-Bs must evade synapsis with chromosome of origin and provide avoidance of pachytene checkpoint. This could be obtained by following the route of sex chromosomes which is indicated by positioning in their vicinity. However, in their distal part, Bs contain some “admixture” of other DNA repeats, homologous to pericentromeric heterochromatin regions of autosomes. It was demonstrated by FISH analysis and the published data on the sequencing, which indicate the presence of similar DNA repeats in Bs of *A. flavicollis* and pericentromeric heterochromatic regions in autosomes of the house mouse [11,20]. Thus, we can propose an origin of Bs in *A. flavicollis* from different DNA repeats, localized in pericentromeric regions of sex chromosomes, on the one hand, and autosomes, on the other hand. The process of B-chromosome formation may undergo simultaneously with the participation of both types of DNA repeats or in two or more stages, starting with one type of repeats (that led to a proto-B chromosome appearing) and continuing by adding of the other repeat types. It is probably just one of the possible evolutionary scenarios for the origin of B chromosomes in this species; if amplification of DNA repeats of autosome heterochromatin was the initial stage, the proto-B chromosome might be a microchromosome, which contained repeats, standard for autosome pericentromeric regions. At later stages of its evolution, the addition of DNA repeats, characteristic for heterochromatin of sex chromosomes, to the proto-B chromosome and their amplification led to the emergence of the new extended region that moved the cluster of initial pericentromeric repeats away from the centromere to the distal regions of the long arms of Bs.

Considering the possible mechanisms of the emergence of Bs containing DNA fragments from different chromosomes, we should recall small supernumerary marker chromosomes (sSMC) in human [62] as well as the homogeneously stained regions (HSRs) that form in cancer cells during some oncological diseases [63,64]. In addition to the HSRs formation, multiplication of different genomic regions can occur, resulting in the appearance of multiple copies of double-minute chromosomes (Dms) [65,66]. Like HSRs, they usually contain *C-MYC* gene [67]. Dms could be considered as precursors of Bs, but they have no centromeres. Since they consist of a transcriptionally active material, it is difficult to assume the formation of a neo-centromere with a subsequent increase in their size, similar to the formation of HSR [68]. It is also unlikely that Bs were formed by the propagation of repetitive sequences in the euchromatic part of chromosome arms, accompanied by the appearance of duplications clusters. The possibility of such a mechanism for the emergence of Bs has been recently proposed and discussed [16]. However, in this case, the pericentromeric repeats should retain their localization.

We also cannot exclude the formation of Bs in *A. flavicollis* from sex chromosomes only. Most of the distal part of the pericentromeric C-positive region of the X chromosome was possibly formed due to amplification of autosome pericentromeric DNA repeats and their translocation, while the proximal area of X chromosome heterochromatin region is represented by specific repeats, characteristic only for sex chromosomes. In the Y chromosome, the proximal region is enriched with DNA repeats homologous to autosome pericentromeres, and the distal region contains repeats, typical for sex chromosomes. So, it is quite possible that only sex chromosomes might take part in arising of Bs—taking into account the FISH data, which indicate the heterogeneity of DNA clusters in pericentromeric heterochromatin of X and Y chromosomes. Moreover, variations in size and DNA composition of C-positive regions of X and Y chromosomes indicate the ongoing processes of DNA amplification and reorganization in their heterochromatin, which may continue to deposit new repeats within Bs. Furthermore, DNA repeats, homologous to DNA of pericentromeric regions of sex chromosomes and the large proximal regions of Bs, were also found in the distal C-positive regions of two pairs of small autosomes (Figure 1, Figure 2 and Figure 3) which implicitly indicates an increased potential for mobility and amplification of these repeats, most of which is accumulated in sex chromosomes. Considering the possible participation of sex chromosomes in the *A. flavicollis* karyotype changes, the whole genome sequencing is of particular interest. The preliminary DNA library sequencing of microdissected B chromosome of *A. flavicollis* revealed the Kdm6 gene [20], which is localized on the X chromosome (in an area, close to the ancestral pseudoautosomal region) in placental mammals [69]. The result confirms our hypothesis that Bs in *A. flavicollis* may have the pseudo-autosomal (i.e., X chromosomal) origin [41].

### 4.2. Comparative Analysis of Bs Spatial Localization in Different Species of Mammals

Spatial organization of the interphase nucleus in the studied individuals of *A. flavicollis* is quite unusual, possibly due to the relatively small size of the C-positive regions in the autosomes. These regions are localized at the periphery of the nucleus as separate elements or in small associations, leaving much free space for C-positive regions of sex chromosomes and Bs. Perhaps for this very reason as well as due to a small number of heterochromatic Bs in *A. flavicollis*, B chromosomes in this species were always localized at the periphery of the interphase nucleus, in the compartment of structural heterochromatin, sometimes, even in the lateral contacts with the nuclear membrane. We did not identify associations between Bs and pericentromeric regions of autosomes in yellow-necked mice. However, problems with the identification of pericentromeric regions of autosomes, which could form an association with one of the sex chromosomes or the B chromosome, do not allow us to conclude that such associations are absent.

In the East-Asian field mouse *A peninsulae* [38], all As contain large C-positive regions: pericentromeric C-positive regions in autosomes, intercalary C-positive block in the proximal region of the X chromosome, and C-positive region in the distal part of the long arm of the Y chromosome. Bs are extremely variable in this species, both in their morphology and size, from dot-like to large [33,57]. The number of Bs in the cells of *A. peninsulae* specimens, studied by us varied from 3 to 19, and the amount of their heterochromatin could be several times greater than the heterochromatin of As. As a result, a significant part of the outer compartment of the interphase nucleus was occupied by large associations of heterochromatic regions of both As and Bs. Interestingly, we unexpectedly observed a non-random distribution of Bs in the associations of the heterochromatic regions. Furthermore, during the formation of chromocenters and conglomerates of centromeric heterochromatic regions of A chromosomes with Bs, their associations in the round nuclei of spermatocytes were higher than in the flat nuclei of fibroblasts [38]. These data indicate a difference in the degree of associations and localization of the autosomal pericentromeric regions between the yellow-necked mouse and the East-Asian field mouse.

Besides the two mouse species, the localization of Bs in the interphase nuclei of mammalian cells has so far been studied only in the fox and the raccoon dog [70,71]. The small B chromosome in the fox cells was located preferentially in the inner compartment of the interphase nucleus, while the larger B chromosome of the raccoon dog was located preferentially in the outer compartment of the interphase nucleus [70]. These authors believe that the results are in good agreement with the hypothesis of the size-dependent localization of chromosomes in the interphase nucleus. According to the hypothesis, in mammals, the most typical organization of the nucleus includes localization of chromosomal heterochromatic regions in the nuclear periphery near or in contact with the lamina and also around the nucleoli, whereas chromosomal regions containing transcriptionally active genes are mostly located in the internal compartment of the nucleus [27,55,72]. Our analysis of spatial localization of chromosomal territories of Bs of *A. flavicollis* showed that they are located predominantly on the border of the nucleus, similarly to pericentromeric regions of autosomes. It seems that structural organization of Bs may be the main factor defining the localization of Bs in interphase nuclei and that can explain different position in different species. Additionally, different type of Bs could have different positions in the same species. This agrees with literature data that chromatin with low content of active genes is predominantly located on the nuclear periphery, near the nuclear lamina and also near the nucleolus. Later, it was noted that the data also well agree with the concept that the location of chromosomes in the interphase nucleus depends on the number and transcriptional activity of their genes [73,74,75]. In addition to repeated DNA sequences, Bs of all these species contain DNA fragments, homologous to the unique coding genes [11], although Bs localize in different compartments of the interphase nuclei. On the other hand, despite the differences in size, composition of repetitive and “unique” sequences (micro-Bs contained only a part of the “unique” sequences, found in macro-Bs, see [11,20]), Bs of the East-Asian field mouse in the interphase nuclei were involved in associations with C-positive regions, located in its outer compartment. These preliminary facts do not confirm the concept. The complete sequence reconstruction for Bs can resolve the problem, but the analysis was not undertaken yet. It should be noted that B chromosome of the raccoon dog contains a large heterochromatic region. Thus, a large C-positive region in the B chromosome of the raccoon dog can determine its peripheral localization since all C-positive regions are located either on the nuclear periphery or around the nucleolus in most cell types [54,74,76,77]. In *A. flavicollis*, heterochromatin of Bs takes part in their association with nuclear membrane too. It is proposed [78] that the formation of the nuclear membrane at the end of mitosis starts with the C-positive chromosomal regions. This may be one of the factors, affecting the localization of heterochromatin of all chromosomes, including Bs, at the periphery of the nucleus in *A. flavicollis* and the raccoon dog. Variation in number and size of C-positive chromosomal regions, including repetitive DNA and variation in the centromere number, might affect the architecture of the interphase nucleus. In the interphase nucleus, the pericentromeric C-positive regions of chromosomes usually form chromocenters, but not in *A. flavicollis*. Thus, the origin and structural peculiarities of heterochromatin of Bs may be the main factor, defining the localization type of Bs in cell nuclei of different species.

## 5. Conclusions

Thus, regardless of the origin of B chromosomes and variability of their DNA composition in them, Bs in different species are predominantly localized into nucleus compartments containing constitutive heterochromatin. Perhaps, degeneration of the DNA repeats is accompanied by a gradual divergence of repeat clusters and their replacement by new DNA repeats into additional chromosomes. The question about the mechanisms of molecular evolution of B chromosomes remains open. The solution of this problem requires a comparison of data obtained from different groups of animals with similar karyotype features.

## Figures and Tables

**Table 1:** Information about *Apodemus flavicollis* and *A. peninsulae* specimens involved in the study.

Species	Collection Number of Specimen, Sex	Karyotype	Cells for Metaphase Chromosome Preparation	Locality of Specimen Collection	Libraries	Remark
*A. flavicollis*	3656, ♀	50,XX,+2B	Cells of bone marrow	Milošev Do, Serbia		used for FISH
	3727, ♂	49,XY,+1B	Gonad tissue	Milošev Do, Serbia	WCP3727B	used for microdissection of B-like chromosomes
	3795, ♂	51,XY,+3B	Cells of bone marrow	Bosilegrad, Serbia		used for FISH
	3854, ♀	51,XX,+3B	Cells of bone marrow	Misača, Serbia		used for FISH 3D-FISH
	3977, ♀	49,XX,+1B	Primary cultured fibroblasts	Petnica, Serbia	WCP3977B	used for FISH
	3978, ♂	48,XY	Primary cultured fibroblasts	Orašac, Serbia		used for FISH
	3979, ♂	48,XY	Primary cultured fibroblasts	Orašac, Serbia		used for FISH
	3980	49,XX,+1B	Primary cultured fibroblasts	Orašac, Serbia	WCP3980B	used for microdissection of B-like chromosomes and for 3D-FISH
	24448, ♂	49,XY,+1B	Cells of bone marrow	Novgorod region, Soletsky district, 60 km S from Veliky Novgorod, bank of Shelon river, Russia		used for S-FISH
	24123, ♂	48,XY		Penza region, Belinsky district, vicinities of Shiryaevo village, Russia		used for FISH
	24943, ♂	49,XY,+1B	Cells of bone marrow	Minsk region, vicinities of Sadovy train station, Republic of Belarus		used for FISH
	24944, ♂	49,XY,+1B	Cells of bone marrow	Minsk region, vicinities of Sadovy train station, Republic of Belarus		used for 3D-FISH
	24985, ♀	51,XX,+3B	Cells of bone marrow	Rostov-on-Don region, Donskoy national park, Russia	WCP24985Ba WCP24985Bb	used for microdissection of B-like chromosomes and for FISH
	26368, ♀	49,XX,+1B	Cells of bone marrow	Ulyanovsk region, Kuzovatovsky district, vicinities of the Naleyka railway station, Russia	WCP26368B	used for FISH
	1851	48,XX	Cells of bone marrow	Novgorod region, vicinities of the town of Malaya Vishera, Russia	PCPAflaCEN	used for FISH
*A. peninsulae*	*A. peninsulae* # 5, ♂	54,XY,+6B	Cells of bone marrow	outskirts of Novosibirsk city, Russia	Ape_mB	used for 3D-FISH
	*A. peninsulae* # 9, ♂	54,XY,+6B micro	Cells of bone marrow	Krasnoyarsk region, near Novokargino settlement, Russia	Ape_dB	used for 3D-FISH

♂ male, ♀ female.

## Data Availability

Raw data would be provided by corresponding authors upon request.
